# Supplementary material for: Empirical methods for controlling false positives and estimating confidence in ChIP-Seq peaks
Source: BMC Bioinformatics. 2008 Dec 5;9:523. doi: 10.1186/1471-2105-9-523 (PMC2628906; doi:10.1186/1471-2105-9-523)
Supplement: Additional File 1 — A variety of html documents from the USeq web site detailing the available applications, their best usage, output file type descriptions, command line menus, etc. [file 1471-2105-9-523-S1.zip › USeqUserGuides/JavaDoc.zip!/JavaDoc/trans/roc/package-use.html]

Uses of Package trans.roc


|  |  |  |
| --- | --- | --- |
| **Overview** \| **Package** \| Class \| **Use** \| **Tree** \| **Deprecated** \| **Index** \| **Help** | |  |
| PREV   NEXT | **FRAMES**    **NO FRAMES**     **All Classes** |  |


---


## **Uses of Package trans.roc**

| Packages that use trans.roc | |
| --- | --- |
| **trans.graphics** | For graphically representing '.cel' files and intervals. |
| **trans.misc** | Rough working scripts. |
| **trans.roc** | For manipulating the BAC dmel spike in data and generating ROC curves. |

Classes in trans.roc used by trans.graphics

****Positive****
  
          Container for a positive BAC used by the Sgr scripts.

****Sgr****
  
          Sgr line container.

Classes in trans.roc used by trans.misc

****Gr****
  
          Gr line container.

****Sgr****
  
          Sgr line container.

Classes in trans.roc used by trans.roc

****Bin****
  
          Score collector for Sgr apps.

****ControlGene****

****Gr****
  
          Gr line container.

****Positive****
  
          Container for a positive BAC used by the Sgr scripts.

****RocWindow****
  
          For holding information about a region for ROC analysis.

****Sgr****
  
          Sgr line container.

---


|  |  |  |
| --- | --- | --- |
| **Overview** \| **Package** \| Class \| **Use** \| **Tree** \| **Deprecated** \| **Index** \| **Help** | |  |
| PREV   NEXT | **FRAMES**    **NO FRAMES**     **All Classes** |  |


---
